# Preemptive Diagnosis of Alzheimer's Disease in the Eastern Province of Saudi Arabia Using Computational Intelligence Techniques

**DOI:** 10.1155/2022/5476714

**Published:** 2022-08-23

**Authors:** Sunday O. Olatunji, Aisha Alansari, Heba Alkhorasani, Meelaf Alsubaii, Rasha Sakloua, Reem Alzahrani, Yasmeen Alsaleem, Reem Alassaf, Mehwash Farooqui, Mohammed Imran Basheer Ahmed, Jamal Alhiyafi

**Affiliations:** ^1^College of Computer Science and Information Technology, Imam Abdulrahman Bin Faisal University, P.O. Box 1982, Dammam 31441, Saudi Arabia; ^2^Department of Computer Science, Kettering University, Flint, Michigan 48504, USA

## Abstract

Alzheimer's Disease (AD) is a silent disease that causes the brain cells to die progressively, influencing consciousness, behavior, planning ability, and language to name a few. AD increases exponentially with aging, where it doubles every 5-6 years, causing profound implications, such as swallowing difficulties and losing the ability to speak before death. According to the Ministry of Health in Saudi Arabia, AD patients will triple by 2060 to reach 14 million patients worldwide. The rapid rise of patients is caused by the silent progress of the disease, leading to late diagnosis as the symptoms will not be distinguished from normal aging affect. Moreover, with the current medical capabilities, it is impossible to confirm AD with 100% certainty via specific medical examinations. The literature review revealed that most recent publications used images to diagnose AD, which is insufficient for local hospitals with limited imaging capabilities. Other studies that used clinical and demographical data failed to achieve adequate results. Consequently, this study aims to preemptively predict AD in Saudi Arabia by employing machine learning (ML) techniques. The dataset was acquired from King Fahad Specialist Hospital (KFSH) in Dammam, Saudi Arabia, containing standard clinical tests for 152 patients. Four ML algorithms, namely, support vector machine (SVM), k-nearest neighbors (k-NN), Adaptive Boosting (AdaBoost), and eXtreme Gradient Boosting (XGBoost), were employed to preemptively diagnose the disease. The empirical results demonstrated the robustness of SVM in the pre-emptive diagnosis of AD with accuracy, precision, recall, and area under the receiver operating characteristics (AUROC) of 95.56%, 94.70%, 97.78%, and 0.97, respectively, with 13 features after applying the sequential forward feature selection technique. This model can assist the medical staff in controlling the progression of the disease at low costs.

## 1. Introduction

Alzheimer's disease (AD) is a prevalent form of dementia in which brain cells progressively die, influencing consciousness, behavior, planning ability, language, and others [1]. It develops silently over a long period that may range from three to ten years before reaching a high level of severity [2]. This may lead to late diagnosis as the symptoms will not be distinguished from normal aging affect. Additionally, AD patients are significantly expected to suffer from several chronic diseases compared to healthy older adults [3]. The authors of [4] claimed that the AD infection rate increases exponentially with aging and doubles every 5-6 years. The Ministry of Health in Saudi Arabia mentioned that 130 thousand of the population were diagnosed with AD in 2020. The Ministry of Health also anticipated that the patients' number would triple by 2060 to reach 14 million patients worldwide [5]. The resources demanded to deliver the best nursing services for the patients which is tremendous and cost-intensive [6]. As a result, numerous researches were conducted to investigate potential treatment procedures to cure or maintain the complication of AD. Most of these studies concluded the importance of early diagnosis and its relevance to executing the optimal treatment plan [7]. Furthermore, according to the World Health Organization (WHO), the development of the disease was proven to be connected with environmental factors [6]. Consequently, this study aims to establish an AD early predictive assistant by employing machine learning (ML) techniques on a Saudi dataset obtained from King Fahad Specialist Hospital.

Currently, it is impossible to confirm AD with 100% certainty via specific medical examinations. Screening and other available diagnostic tools used to identify patients with AD are expensive and not suitable for hospitals with limited resources [6]. Therefore, involving the latest technologies such as ML can assist doctors in achieving a more accurate and efficient diagnosis. With the increasing amount of medical data and the evolving capabilities of ML, physicians will be able to identify hidden patterns that humans are incapable of detecting [8]. Therefore, it can significantly improve the AD patients' lifestyle and boost their prevention chances. Considering Saudi Arabia's recent principle of employing the latest technology, numerous studies were conducted to support the health industry. Researchers in reference [9] obtained a dataset from King Abdulaziz University (KAU) Hospital, Saudi Arabia, to diagnose AD earlier using cerebral catheter angiogram neuroimaging, achieving an accuracy of 99.14%. Furthermore, researchers in references [10–13] utilized various ML techniques to perform a pre-emptive diagnosis of diabetes mellitus, chronic kidney disease, schizophrenia, and thyroid cancer, attaining the highest accuracies of 98.00%, 98.00%, 90.70%, and 90.91%, respectively. The promising results of these studies have encouraged us to consider expanding the work to build a prediction model for AD using simple clinical and demographical data.

As health care began to adopt ML, several algorithms were established to achieve adequate results. In this study, a total of four ML algorithms were utilized, namely, support vector machine (SVM), k-nearest neighbor (k-NN), Adaptive Boosting (AdaBoost), and eXtreme Gradient Boosting (XGBoost), each with robust characteristics. Aside from SVM's flexibility in addressing classification problems, it has been used recently to diagnose precision psychiatry, brain disorders, and many others achieving superior performance [14]. Conversely, k-NN is well known for its simplicity, rapidity, and scalability in handling complex data [15], while AdaBoost is also popular due to its high compatibility and low complexity [16]. XGBoost exceeds AdaBoost in terms of scalability and speed. This method leverages parallelized techniques to accelerate computation by ten times [17].

Empirical results demonstrated that SVM outperformed all the other classifiers achieving an accuracy of 95.52%, followed by k-NN, AdaBoost, and then XGBoost. With only six features and a negligible difference in accuracy, k-NN exceeded the performance of SVM by reducing the required number of clinical tests. However, it is crucial to consider a higher recall value that focuses on reducing false negatives as the misdiagnosis of AD induces severe complications. Hence, it is concluded that the optimal model for pre-emptively diagnosing AD is SVM attaining the highest recall of 97.78% and an area under the receiver operating characteristics (AUROC) of 0.97 using 13 features after applying the sequential forward feature selection technique.

The remaining sections in this paper are arranged as follows.  Section 2 involves the reviewed literature, whereas Section 3 includes the technical description of the four utilized machine learning algorithms. The empirical study is demonstrated in Section 4, containing dataset description, statistical analysis, an experimental setup, performance measures, and an optimization strategy.  Section 5 illustrates and discusses the experiment outcomes, while the last section presents the conclusion and future work recommendations.

## 2. Review of Related Literature

Several publications utilized various ML and deep learning (DL) algorithms to develop early diagnosis models for AD. Below are some of the literature reviews conducted to identify the gaps in the studies and observe the optimal classifiers.  Table 1 represents a summary of the literature reviews with their major limitations.

Alzheimer's Disease Neuroimaging Initiative (ADNI) provides several datasets with different data types for researchers to benefit from while employing ML and DL techniques [31]. The authors in reference [18] utilized the ADNI dataset to enhance the AD prediction accuracy by using the VGG-16 architecture convolution neural network (CNN) model for feature extraction. The empirical results showed that SVM, linear discriminate, and k-NN achieved the highest accuracy of 100% when trained using the functional magnetic resonance imaging (fMRI) images, while k-NN attained the highest accuracy of 76.56% when trained using the positron emission tomography (PET) images.

In another study [19], the authors developed a novel architecture for the early prediction of AD. The proposed 3DMgNet architecture was trained with two magnetic resonance images (MRI) datasets. The first was obtained from the ADNI database, and the other was collected from a hospital located in China. The empirical results revealed that the intended model achieved the highest classification accuracy of 92.133%.

Similarly, the study [20] utilized the ADNI dataset to diagnose AD using three ML classifiers. The features in the dataset were standardized and normalized. The authors concluded that logistic regression (LR) achieved the best results with accuracy, specificity, and sensitivity of 98.12%, 95%, and 90%, respectively.

The authors in reference [21] also used one of the ADNI datasets to propose a state-of-the-art technique that automates the early detection of AD's three stages. Various methods were employed for feature extraction, followed by using the principal component analysis (PCA) to reduce the number of features. The empirical results showed that random forest (RF) achieved the highest accuracy of 98.42%. The study [22] also proposed prediction and detection tools for the early diagnosis and classification of AD using MRI. The CNN algorithm was employed to classify 1512 mild, 2633 normal, and 2480 AD patients, achieving a 99% accuracy.

In another study [23], the authors proposed a technique for detecting AD early using blood plasma proteins. The dataset was acquired from the ADNI portal, containing 146 blood plasma proteins from three clinical groups. A correlation-based feature selection technique was used on the first dataset based on brute force search to form feature panels. Ten-fold cross-validation was used on the first dataset. Then, the resulted models were tested with the second dataset. The empirical results showed that SVM achieved the best results with a sensitivity higher than 80%, specificity above 70%, and at least 0.80 for AUROC for both datasets.

Similarly, using ML, the authors in reference [24] used psychological parameters including education, age, number of visits, and the mini mental state examination to predict AD. The empirical results indicated that SVM achieved the highest accuracy of 85%.

Open Access Series of Imaging Studies (OASIS) datasets were also employed in several studies [32]. The authors in reference [25] detected AD in its early stages using a dataset obtained from OASIS, containing MRI data of 373 imaging sessions for 62 men and 88 women. Feature selection was applied through the Boruta algorithm. The results indicated that RF achieved the best performance among the 12 proposed models with accuracy, sensitivity, specificity, and AUROC of 94.39%, 88.24%, 100.00%, and 94.44%, respectively.

Furthermore, the authors in reference [26] used a data augmentation method to collect AD braining images to implement a robust computer-vision-based prediction model using an 8-layer CNN with leaky ReLU and max pooling. The model attained an accuracy, sensitivity, and specificity of 97.65%, 97.96%, and 97.35%, respectively.

On the other hand, the proposed method in the study [27] used the extracted spectrogram features from speech data to detect AD's early stages. The technique utilized two datasets, where the first dataset consisted of 36 subjects, while the second dataset consisted of 32 subjects. LR-CV was proved to be the best classification model in both datasets. The results after parameter optimization reached a precision, recall, F1 score, and accuracy of 87.5%, 91.3%, 89.4%, and 86.1%, respectively.

The authors in reference [28] utilized four ML algorithms to classify AD patients. Three datasets were used, with each dataset involving 28 mild AD or cognitive impairment patients and 50 cognitively healthy adults. Five-fold cross-validation and leave-one-out were used for evaluation. Moreover, SelectKbest was employed for feature selection, where SVM attained the best outcomes with accuracy, specificity, and sensitivity of 91.08%, 94%, and 85.71%, respectively.

More recently, the authors in reference [29] used clinical data for AD early prediction. To increase the prediction accuracy, two-stage categorization techniques were applied. The first stage entailed investigating the impact of diabetes and high blood pressure on the cognitive decline; consequently, they trained SVM and RF. After that, the cognitive function with possible dementia was examined in the second step utilizing a neuropsychological test called the cognitive ability test (CAT). The multinomial LR approach was used to analyze CAT findings to forecast the likelihood of cognitive deterioration later in life. The proposed work achieved an accuracy of 0.86 with SVM for the first stage, while RF attained an accuracy of 0.71 for the same stage. Multinomial LR attained an accuracy of 0.89 for the second stage.

Furthermore, 3D-CNN was utilized in reference [30] for the early prediction of AD. The dataset was taken from the MIRIAD dataset, and it contained MRI scans of 46 AD and 23 non-AD controls. The 3D-CNN model achieved a testing accuracy of 100% with a loss of 12.74%, validation accuracy of 98.08% with a loss of 14.59%, and training accuracy of 100% with a loss of 9.5%, and precision, recall, and F1 score of 100%.

Based on the literature reviews related to this study, it was observed that several classifiers achieved good performance, such as conventional neural networks (CNN), support vector machine (SVM), random forest (RF), and logistic regression (LR). It was also observed that most of the reviewed papers utilized imaging datasets, which increased the workload of collecting data and the inconvenience of using high-complexity assembled models by nontechnical individuals. Additionally, it was noted that most of the studies achieved low sensitivity rates, whereas other studies achieved insufficient classification accuracy. To overcome these limitations, this work aimed to construct a model using simple clinical features and obtain high accuracy and recall rates with minimal computation time using a dataset collected from a Saudi hospital. Therefore, local hospitals with limited equipment can benefit from the pre-emptive diagnosis models. In addition, it sought to resolve the problem of imbalanced data that may reduce sensitivity by applying the Synthetic Minority Oversampling Technique (SMOTE).

## 3. Materials and Methods

**Figure 1:**
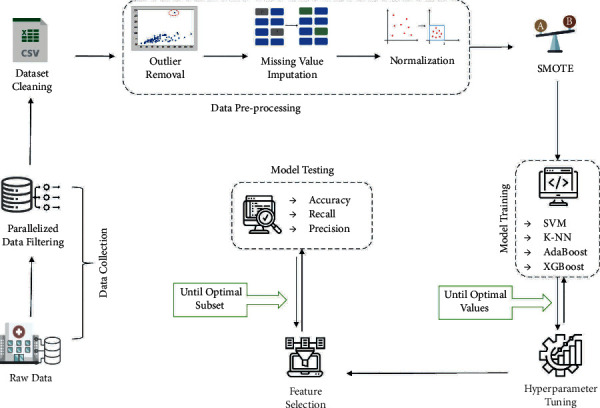
The study framework.

Python programming language was utilized in this experiment to develop the pre-emptive diagnosis models for AD using Google Colaboratory. The Sklearn library was utilized to perform data preprocessing and modeling. Four ML algorithms were used, including support vector machine (SVM), eXtreme Gradient Boosting (XGBoost), k-nearest neighbor (k-NN), and Adaptive Boosting (AdaBoost). Other steps, including balancing the dataset, optimizing the algorithms using GridSearchCV, and feature selection using Sequential Forward Feature Selection, were performed using the Sklearn and Mlxtend libraries with stratified 10-folds cross-validation. The random state was set to 0 for all the implemented steps.  Figure 1 illustrates the study's framework, and the following sections explain the flow of the study in detail.

### 3.1. Dataset Collection and Cleaning

The Saudi Alzheimer's disease dataset was obtained from King Fahad Specialist Hospital (KFSH), Dammam, Saudi Arabia. It initially consisted of 11 features and 53522 instances, where it had to be filtered to extract the laboratory test for each patient in a single row. Therefore, a filtering code was proposed for Alzheimer's dataset, where parallelism techniques were employed to reduce the computation complexity. The parallel computing paradigm involved executing several instructions simultaneously to reduce computation complexity and time. The multiprocessing library is one of Python's most commonly used modules to implement parallel computing. It performs process-based parallelism through a fork system call where all processes share the same memory [33]. The module provides various approaches to execute code blocks concurrently, including Process, Pool/Map, Pool/apply_async, and others.

To conduct this experiment, we utilized the Pool method, a class that assembles a pool of processes, along with the map method, which traverses the input element list in parallel [34]. Assuming a system with N processors or nodes, the speedup in comparison with one processor can be estimated as follows:

(1) $$\begin{array}{c} \text{Speedup}=\frac{T_{1}}{T_{N}}, \end{array}$$

where *T*_1_ is the code runtime for one processor and *T*_*N*_ is the runtime for *N* processors. *T*_*N*_ depends on the number of computing nodes and the proportion of serial code. Hence, the total runtime of the parallelized code can be measured using Amdahl's law:

(2) $$\begin{array}{c} T_{N}=T_{S}+\frac{T_{P}}{N}+T_{\text{Sync}}, \end{array}$$

where *T*_*S*_ represents the execution time of the serial code, *T*_*P*_ represents the runtime of the parallel code, and *T*_Sync_ represents the time needed for synchronization operations such as I/O operations [34].

In this study, patients' records in the dataset were organized using the proposed pseudocode presented in Algorithm 1. The map function provided by Python was employed with seven cores instead of the complete number of processors to avoid freezing the program.

As demonstrated in Algorithm 1, we began by defining the dataset, creating the pool, and mapping it to the data filtering function with the list of intended columns. The data filtering function presented in Algorithm 2 traverses through every patient record to fill their information in parallel. After filling the columns, the pool was closed, and the results obtained were transferred to the dataset variable.

### 3.2. Dataset Description

After applying the filter code and removing the duplicate records, the dataset contained standard clinical tests for 152 patients, where 63 were positively diagnosed with AD and 89 were negatively diagnosed with AD. The dataset also incorporated 435 clinical features. However, only 18 attributes were utilized to build the AD prediction model after removing the columns containing missing values exceeding 30% of the total number of instances.  Table 2 outlines the attributes used in this study.

### 3.3. Statistical Analysis

Statistical analysis provides essential tools for visualizing and understanding the data patterns for better preprocessing and modeling.  Table 3 displays the statistical analysis for the Saudi AD dataset's numerical features, including the mean, standard deviation (STD), minimum (Min), first quartile (25%), median (50%), and third quartile (75%), and maximum (Max) values for each feature. Moreover, it outlines the count of missing values in each column.

Statistical analysis tools can show the presence of outliers in the data. For instance, the significant difference between the minimum values and the first quartile of the BP-systolic, respiratory rate, BP-diastolic, and pulse ox attributes shows the presence of outliers. Additionally, the considerable difference between the maximum value and the third quartile of the platelet attribute indicates the presence of an outlier. The standard deviation shows a wide spread of data in the platelet, BP-systolic, pulse, and BP-diastolic. In contrast, it demonstrates a considerably low distribution of the other attributes around the mean.

### 3.4. Dataset Preprocessing

The data fed into ML algorithms require several preprocessing steps to enhance the learning process of the algorithms. In our study, the Sklearn library was utilized to preprocess the data starting by transforming all entries into numerical values, either float or integer, and the platelets values mentioned as “normal limit” were set to 250. The following preprocessing steps included treating outliers, filling the missing values, and scaling the data.

(1) Outlier removal: The outliers were treated by replacing the minimum values of the pulse ox and respiratory rate attributes and the maximum value of the platelet attribute with their mean:
  (3) $$\begin{array}{c} \text{Mean }=\;\frac{\sum X}{n}, \end{array}$$
where *x* denotes the data points and *n* is the number of points.
(2) Missing values imputation: A k-NN imputer replaces missing values with the mean estimation of the nearest neighbor based on a specific similarity measure [35]. In our study, the missing values were treated with the nearest neighbor's value set to 3 and the metric set to Euclidean distance. The k-NN imputer operates as follows:
  (1) Define the n_neighbors.
  (2) Calculate the Euclidean distance:
    (4) $$\begin{array}{c} d\left(p,q\right)=\;\sqrt{\sum_{i=1}^{n}{\left(q_{i}-p_{i}\right)}^{2}}, \end{array}$$
  where *p* and *q* correspond to different points and *n* denotes the n-space.
  (3) Impute the missing values using equation (3).
(3) Data Scaling: The MinMaxScaler operates by scaling the values of the attributes to a specific range. In our study, the attributes were scaled in the range of 0 to 1 as
  (5) $$\begin{array}{c} \text{MinMaxScaler }\left(v^{\prime }i\right)=\;\frac{vi-{\text{min}}_{A}}{{\text{max}}_{A}-{\text{min}}_{A}}\left(\text{new}\_{\text{max}}_{A}-\text{new}\_{\text{min}}_{A}\right)+\text{new}\_{\text{min}}_{A}. \end{array}$$
where *vi* represents the data point, max_*A*_ and min_*A*_ represents the maximum and minimum values of an attribute, and new_max_*A*_ and new_min_*A*_ denotes the values 0 and 1, respectively.

### 3.5. Description of the Utilized Machine Learning Techniques

The sections below represent a theoretical background of the classifiers utilized to pre-emptively predict the possible incidence of Alzheimer's disease.

#### 3.5.1. Support Vector Machine (SVM)

Support vector machine (SVM) is a trendy statistical-based supervised machine learning algorithm introduced in the late 1990s by Vapnik, Cortes, and Boser to solve classification and regression problems [36]. It has a distinctive ability to model complex relationships between variables and merge generalization control with a procedure to address the curse of dimensionality, which arises when a model examines a dataset with various features and limited samples, allowing the algorithm to perform superbly well with limited instances [14]. For linearly separable data, the SVM algorithm explores the training instances belonging to a particular class and then constructs a boundary that divides the training data into two separated classes. This boundary is known as the hyperplane, which is a subspace with dimension p-1 [37]. Equations (6) and (7) represent the formulas for finding the maximum margin hyperplane, where $\overset{⟶}{w}$ represents a vector of *m* dimensions, *b* denotes a scalar, *g* represents the slack variable, *c* denotes the penalty parameter, and *y*_*i*_ is the class label [38].

(6) $$\begin{array}{c} \text{min}:P\left(\overset{⟶}{w},b,g\right)=\frac{1}{2}{\overset{⟶}{w}}^{T}.\overset{⟶}{w}+c\sum_{i=1}^{l}g, \end{array}$$

(7) $$\begin{array}{c} \text{subject to}:\left\{\begin{array}{c} y_{i}\left({\overset{⟶}{w}}^{T}\emptyset \left({\overset{⟶}{x}}_{i}\right)\right.+b\geq 1-g,\\ g\geq 0,\;i=1,2,\ldots ,l. \end{array}\right. \end{array}$$

In order to find the optimal hyperplane, the margin, which is the distance between the hyperplane and support vectors, should be maximized. On the other hand, the separation of nonlinear data is enabled by introducing the kernel functions, where the SVM algorithm utilizes a kernel trick that finds an optimal boundary for the probable outputs.

#### 3.5.2. K-Nearest Neighbor (K-NN)

K-nearest neighbor (K-NN) is a supervised machine learning algorithm developed by Evelyn Fix and Joseph Hodges in 1951 and then expanded by Thomas Cover [39]. It is often referred to as a nonparametric, instance-based, memory-based, and lazy learner technique as each term represents a characteristic of K-NN. The principle of this classifier concentrates on using the nearest data points (neighbors) to predict classes or continuous values for hidden data. The quality of the predictions depends on the distance measurement. Executing k-NN begins with recognizing the number of neighbors (*K*) that have the minimum distance between the data points and the target point. Subsequently, a majority voting mechanism is implemented to choose an appropriate class or value for the new data. Equation (8) represents the Minkwiski distance measure that can be generalized to calculate other distance measures:

(8) $$\begin{array}{c} d\left(x,y\right)={\left(\overset{k}{\underset{i=1}{\sum }}{\left(\left|x_{i}-y_{i}\right|\right)}^{p}\right)}^{1/p}, \end{array}$$

where *x* and *y* correspond to different points and *p* turns the formula to Manhattan distance if set to 1 and Euclidean distance if set to 2.

#### 3.5.3. Adaptive Boosting (AdaBoost)

Adaptive Boosting (AdaBoost) is an ensemble technique that implements boosting to construct a robust algorithm from a combination of weak algorithms in series. This method was first introduced by Freund and Schapire in 1997 [16]. It is mainly used to enhance the decision tree's performance for classifying binary problems. In the training process, each model concentrates specifically on the weakness of the previous model by adjusting the weights of the data points. The misclassified samples' weights will be boosted, whereas the weights of the accurately classified samples will be decreased. After applying the same procedure to each model, the results will be aggregated into a robust classifier [16]. The steps for the AdaBoost algorithm are explained briefly along with the mathematical equations below.

The dataset is represented in the following equation, where *n* represents the number of features, *x* is the set of instances, and *y* is the binary target attribute:

(9) $$\begin{array}{c} x_{i}\in {\mathbb{R}}^{n},\;y_{i}\in \left\{-1,1\right\}. \end{array}$$

All instances have the same weighted sample *w* at the beginning, where N is the total number of instances:

(10) $$\begin{array}{c} w=\frac{1}{N}\in \left[0,1\right]. \end{array}$$

Then, the classifier's influence in classifying the instances is calculated using the equation below, where *α* represents the influence and TotalError represents the error rate:

(11) $$\begin{array}{c} \alpha =\frac{1}{2}\ln\frac{\left(1-\text{TotalError}\right)}{\text{TotalError}}. \end{array}$$

After calculating *α*, the sample weights are updated using the following equation, where *α* is positive if the instance was classified correctly and *α* is negative if it was misclassified:

(12) $$\begin{array}{c} w_{i}=\;w_{i-1}*e^{\pm \alpha }. \end{array}$$

The sample weight will be reduced if the classification was accurate and increased if it was inaccurate to avoid misclassification repetitions [40].

The final equation for AdaBoost classification is demonstrated below, where *h* represents the weak classifier and *α* represents its corresponding weight [41]:

(13) $$\begin{array}{c} H\left(X\right)=\text{sign }\left(\sum_{t=1}^{T}\alpha_{t}h_{t}\left(x\right)\right). \end{array}$$

#### 3.5.4. eXtreme Gradient Boosting (XGBoost)

eXtreme Gradient Boosting (XGBoost) is a robust ensemble classifier introduced in 2014 by Chen and Guestrin to solve classification and regression problems [42]. It is extended from the gradient boosting trees, which trains trees sequentially to reduce the error of the prior model. The output $\hat{y}$ is calculated by summing the scores predicted by trees. Equation (14) represents the formula for calculating the outcome [43]:

(14) $$\begin{array}{c} \hat{y}=\sum_{k=1}^{K}f_{k}\left(x_{i}\right),\;f_{k}\epsilon F, \end{array}$$

where *k* represents the number of trees, *f*_*k*_(*x*_*i*_) denotes the *k*^*th*^ tree's score, and *F* is the function's space compromising the trees.

XGBoost refines the gradient boosting trees by introducing several algorithmic enhancements. It is capable of improving the convergence speed of the model's training by utilizing the second derivative and performing the second-order Taylor expansion, allowing more profitable model exploration and efficient usage of memory resources. Additionally, XGBoost effectively reduces the overfitting problem by employing regularization techniques and introducing column sampling. Equation (15) represents the formula for optimizing the objective function by adding the regularization technique [44]:

(15) $$\begin{array}{c} \text{obj}\left(\theta \right)=\sum_{i=1}^{n}l\left(y_{i},{\hat{y}}_{i}\right)+\sum_{k=1}^{K}\Omega \left(f_{k}\right), \end{array}$$

where *l* denotes the loss function, $\sum_{i=1}^{n}l\left(y_{i},{\hat{y}}_{i}\right)$ the differentiable loss whereas ∑_*k*=1_^*K*^*Ω*(*f*_*k*_) signifies the regularization technique added.

### 3.6. SMOTE

The Synthetic Minority Oversampling Technique (SMOTE) is an oversampling approach introduced by Chawla, Bowyer, Hall, and Kegelmeyer in 2002. It is considered one of the most effective data sampling techniques in classification tasks due to its simplicity and robustness. SMOTE aims to reduce data imbalance in the training data and enhance models' generalization on the testing data. It operates by generating synthetic samples of the minority class to make them approximately equal to the majority class's samples [45]. An abstract of the method is shown in equation (16), where *x*_*i*_ is a minority class's sample, *x*_*i*_^*p*^ are randomly chosen samples of its nearest neighbors with *p*=1,…*N*, *u* is a random value between [0, 1], and *x*_*i*_^*∗p*^ is the new synthetic sample:

(16) $$\begin{array}{c} x_{i}^{*p}=x_{i}+u\left(x_{i}^{p}-x_{i}\right). \end{array}$$

The previous steps are applied when the features contain numeric values. In the case of nominal values, one of two values is selected at random.

## 4. Establishment of Alzheimer's Disease Prediction Model

### 4.1. Performance Measures

This study employed three performance measures: accuracy, precision, and recall to evaluate the models' performance. Precision computes the number of true positive predictions that belong to the positive class:

(17) $$\begin{array}{c} \text{Precision}=\;\frac{\mathrm{TP}}{\mathrm{TP}+\mathrm{FP}}. \end{array}$$

Recall calculates the number of true positive predictions assembled out of all positive examples:

(18) $$\begin{array}{c} \text{Recall}=\;\frac{\mathrm{TP}}{\mathrm{TP}+\mathrm{FN}}. \end{array}$$

Accuracy is the primary performance measure that assesses the correct number of predictions. Equation (19) presents the mathematical representation for calculating the accuracy:

(19) $$\begin{array}{c} \text{Accuracy}=\;\frac{\mathrm{TP}+\mathrm{TN}}{\mathrm{TP}+\mathrm{TN}+\mathrm{FP}+\mathrm{FN}}, \end{array}$$

where true positive (TP) indicates the instances classified with AD correctly, false positive (FP) represents the instances classified with AD incorrectly, true negative (TN) denotes the instances classified with nonAD correctly, and false negative (FN) presents the instances classified with nonAD incorrectly.

In addition to the aforementioned performance measures, the receiver operating characteristics curve (ROC) was plotted to measure the accuracy in the sense of the capability to compare the performance of the classifiers across the whole range of class distributions. In other words, the ROC curve measures how well a model can differentiate between two objects [46].

### 4.2. Optimization Strategy

Performing hyperparameter optimization mechanisms helps in improving the model to obtain better results. Therefore, the GridSearchCV with stratified 10-folds cross-validation has been utilized to identify the optimal hyperparameter to obtain the highest possible accuracy for each model. GridSearch operates by trying all the hyperparameter values in all combinations to produce the most accurate outputs.

The hyperparameter grid for SVM consisted of cost, gamma, and kernel. The range of cost values was between 1 and 30. The gamma included the values {1, 0.1, 0.01, 0.001, 0.0001}. Additionally, the grid included the kernel functions {RBF, Sigmoid, Linear}.

The hyper-parameter grid for k-NN consisted of n_neighbors and metrics. The range of the n_neighbors included the odd numbers between 5 and 39. Additionally, the grid included the metrics {Minkowski, Euclidean, Manhattan}.

The hyperparameter grid for AdaBoost consisted of N_estimators and learning rate. The range of the N_estimators values included {100, 200, 300, 400, 500, 600, 700, 800, 900, 1000} and learning rate values included {0.001, 0.01, 0.1, 0.0001}.

The hyperparameter grid for XGBoost consisted of N_estimators, Booster, and learning rate. The range of the N_estimators values included {50, 100, 200, 300, 400, 500, 600, 700, 800}. The booster values included {Gbtree, Gblinear, Dart}. Furthermore, learning rate included the values {0.0001, 0.001, 0.01, 0.1}.

Table 4 outlines the optimal hyperparameters produced by the GridSearchCV algorithm applied using the original and oversampled datasets to compare their results.

## 5. Results and Discussion

To assess the utility of performing SMOTE, the GridsearchCV algorithm was applied to the algorithms while training them on the sampled and original data to obtain the optimal hyperparameters for each experiment. Stratified 10-folds cross-validation was utilized to assess the models' performance in terms of the previously outlined performance measures.  Table 5 compares the results of the proposed models before and after applying the SMOTE technique.

The results demonstrate an inconsequential difference in the accuracy and precision before and after applying the SMOTE algorithm. In contrast, they reveal a significant difference in the recall after applying the SMOTE sampling technique. This is a consequence of the disproportion between the number of positive and negative patients in the dataset, in which the models are biased toward predicting the negative class correctly and mispredicting the positive class. Since AD is a critical disease that must be identified pre-emptively to avoid further complications, it is necessary to consider the increment in the recall rate that focuses on reducing the number of FN. Therefore, it is concluded that the SMOTE algorithm positively impacted the performance of the models, where SVM attained the best outcomes with an accuracy of 93.33%, precision of 93.32%, and recall of 95.56%. The subsequent experiments were conducted with the models trained on the sampled data.

### 5.1. Feature Selection

Feature selection plays a vital role in reducing the computational power of building models as it dismisses irrelevant features that can negatively impact a model's performance. In this study, the Sequential Forward Feature Selection algorithm was utilized to automatically choose the feature subset that results in the best performance for each algorithm [47]. The Sequential Forward Feature Selection algorithm operates by training an algorithm using the best feature decided by a specific criterion function. In each sequential forward step, the feature that improves the accuracy is added to the features subset as represented by Algorithm 3.  Table 6 exhibits the best feature subset yielded by the Sequential Forward Feature Selection algorithm.

The authors in reference [48] aimed to investigate the significance of two feature selection techniques, namely, principal component analysis (PCA) and linear discriminant analysis (LDA). It was concluded that datasets with low dimensionality produce better results when ML algorithms are used without dimensionality reduction. Despite the low dimensionality of the dataset, our study showed that the Sequential Forward Feature Selection algorithm improved the ML algorithms' performance.

It is concluded from Table 6 that SVM achieved the highest accuracy of 95.56% with 13 features, followed by k-NN with an accuracy of 95.52% using six features. Since the difference in accuracy is insignificant, it is concluded that k-NN outperformed SVM in terms of the number of the few clinical tests needed to achieve high accuracy, which can reduce the lab test costs. Additionally, it is indicated that the critical attributes for pre-emptively predicting AD are “age” and “sex” as they were utilized in all models.

### 5.2. Further Discussion of the Results

**Figure 2:**
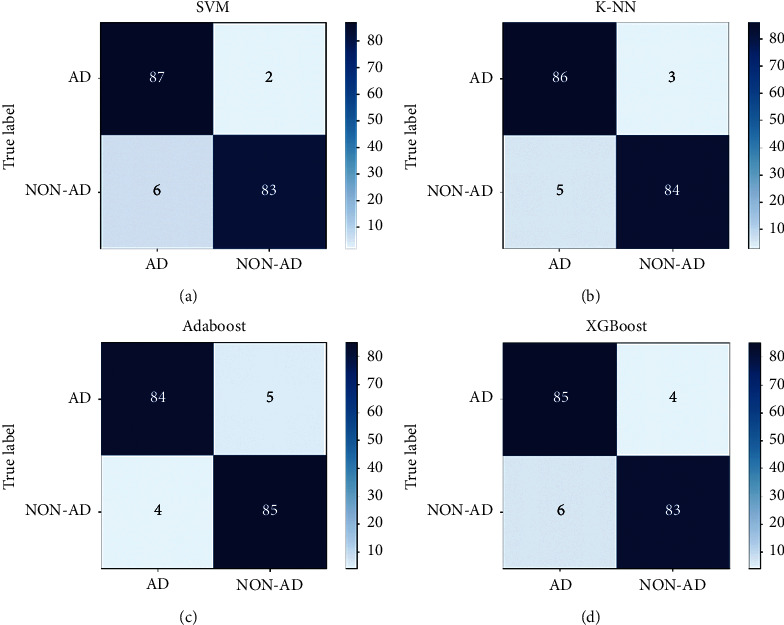
(a) SVM confusion matrix, (b) k-NN confusion matrix, (c) AdaBoost confusion matrix, (d) XGBoost confusion matrix.

Table 7 evaluates the proposed models with the optimal hyperparameters and feature subsets in terms of the previously defined performance measures. It is indicated that SVM achieved the highest accuracy of 95.56%, followed by k-NN almost reaching the same accuracy rate with a difference of only 0.03%. In contrast, XGBoost obtained the lowest accuracy of 94.38%. It is also noted that the highest precision rate of 96% was attained by AdaBoost, while XGBoost achieved the lowest precision rate of 94.18%. Correspondingly, the highest recall rate of 97.78% was achieved by SVM, whereas Adaboost attained the lowest recall rate of 94.44%.  Figure 2 illustrates the confusion matrices of the final selected models.

It is observed from Figure 2 that the lowest FN and FP corresponding values of 2 and 4 were achieved by SVM and AdaBoost, respectively. On the other hand, the highest FN value of 5 was obtained by AdaBoost, whereas SVM and XGBoost attained the highest FP value of 6. As a result of the proposed models achieving similar outcomes, it is challenging to deduce the underperforming model from the observed values. Nevertheless, FN result in the misdiagnosis of Alzheimer's disease leading to severe consequences such as losing the opportunity for early treatment and potentially escalating the disease's progression [49]. Hence, it is critical to attain the lowest possible FN value when considering the best-performing model, determining that SVM outperformed the other models pre-emptively diagnosing Alzheimer's disease.

**Figure 3:**
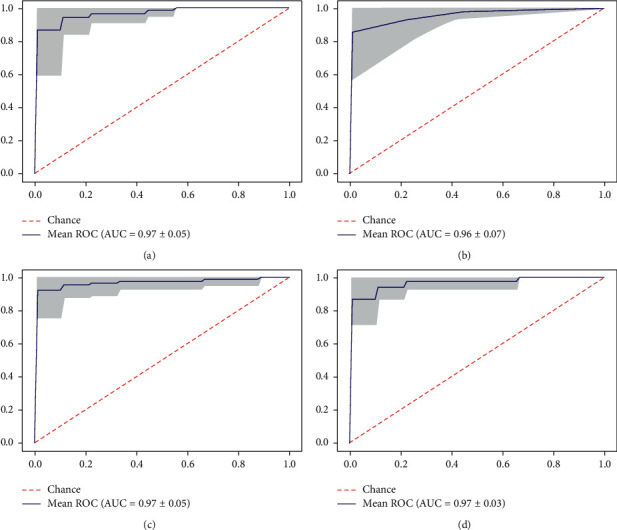
(a) SVM ROC-AUC curve, (b) K-NN ROC-AUC curve, (c) AdaBoost ROC-AUC curve, (d) XGBoost ROC-AUC curve.

Figure 3 demonstrates the Area Under the Receiver Operating Characteristics (AUROC) generated using stratified 10-fold cross-validation to evaluate the models' capability to distinguish between patients with AD and those who do not. It is suggested that SVM, AdaBoost, and XGBoost models attained the same excellent AUROC of 0.97, followed by k-NN with a slightly less AUROC value of 0.96. In general, the achieved results indicate the proposed models' outstanding discrimination ability in producing excellent predictions.

This study aims to employ machine learning algorithms for the pre-emptive diagnosis of AD by utilizing simple clinical data. The study demonstrated SVM's superior performance to the proposed models in respect of accuracy and recall rates of 95.56% and 97.78%, respectively, followed by k-NN with slightly lower accuracy of 95.53% and a lesser recall of 96.67%. Although k-NN achieved comparable accuracy using only six features, leading to fewer clinical tests, which may contribute to reducing the costs, it falls short regarding recall rate. A high recall rate ensures fewer FN presence as the FN predictions lead to severe consequences due to misdiagnosis. For further analysis of the best two models, AUROC was generated to evaluate the ability of the proposed models to classify Alzheimer's patients. SVM outperformed k-NN with an AUROC of 0.97, whereas k-NN obtained a slightly less AUROC of 0.96. Overall, SVM is concluded to be the best-performing model in the early diagnosis of Alzheimer's with superior accuracy, recall, and AUROC.

A delay in AD diagnosis results in rapid disease progression and potentially risks the patients' lives as the disease gradually develops. The utilization of simple clinical data in the proposed models aids in the early diagnosis of Alzheimer's due to its fewer risks and costs compared to MRI scans. For instance, patients with implanted medical devices, such as a pacemaker, are restricted from taking MRI scans due to the danger of burns, undesirable device movements, and device malfunctions. Moreover, MRI scans can potentially pose harm to the patient's body if used inadequately, along with other safety concerns [50]. Therefore, developing an accurate machine learning model using clinical data can benefit hospitals in the pre-emptive prediction of AD at a lower cost.

## 6. Conclusion and Recommendations

This project was undertaken to develop an ML model that pre-emptively predicts AD to enhance the prevention procedures and decrease the mortality rate induced by this illness. Four algorithms were trained using a Saudi dataset that was obtained from King Fahad Specialist Hospital, including support vector machine (SVM), k-nearest neighbor (k-NN), Adaptive Boosting (AdaBoost), and eXtreme Gradient Boosting (XGBoost). Results confirmed the robustness of machine learning in predicting AD using simple clinical features. SVM achieved the best performance among the other classifiers, considering the accuracy of 95.56%, recall of 97.78%, and AUROC of 0.97. Although the number of features required to attain these results was almost double the number needed for k-NN to accomplish an accuracy of 95.53%, the recall value of SVM is prioritized in the medical field. Consequently, future work may include investigating approaches to reduce the number of features while maintaining high accuracy and recall. The experiment can also be expanded to address other chronic disorders. Moreover, it is recommended to use the least computational techniques and datasets available while experimenting with medical problems. This would facilitate adapting the pre-emptive prediction tool in most healthcare facilities while addressing the economic concerns.

## Figures and Tables

**Algorithm 1:**
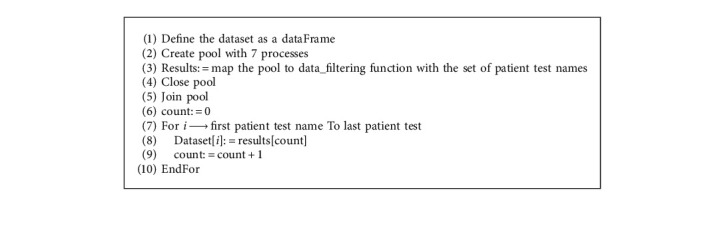
Data filter parallelized pseudo code.

**Algorithm 2:**
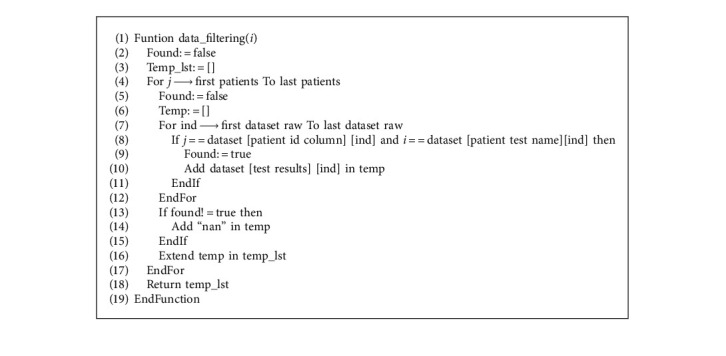
Data filter process pseudo code.

**Algorithm 3:**
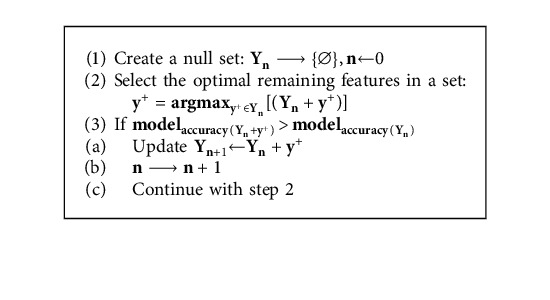
Sequential forward feature selection algorithm.

**Table 1:** Literature reviews summary.

#	Author/s	Technique/s	Results	Limitations
[18]	Janghel and Rathore	Support vector machine (SVM), linear discriminate, and K-means clustering	SVM, linear discriminate, and the K means clustering achieved an accuracy of 100% using FMRI images, while k-NN achieved the highest accuracy of 76.56% using PET images	The datasets utilized are imbalance
[19]	Gao et al.	Novel 3DMgNet architecture	The proposed architecture achieved an accuracy of 92.133%, sensitivity of 88.42%, specificity of 95.00%, and AUC of 94.443	The model's sensitivity is considered low
[20]	Memon et al.	Logistic regression (LR), decision tree (DT), and support vector machine (SVM)	LR achieved an accuracy, specificity, and sensitivity of 98.12%, 95%, and 90%, respectively	The sensitivity is considered low
[21]	Dinu and Manju	Random forest (RF) and tree bagger (TB)	RF achieved an accuracy of 98.42%, sensitivity of 0.85, and specificity of 0.95	The sensitivity is considered low
[22]	Salehi et al.	Convolutional neural network (CNN)	CNN achieved an accuracy of 99%	The dataset is imbalance
[23]	Eke et al.	Support vector machine (SVM)	SVM achieved a sensitivity higher than 80%, specificity above 70%, and at least 0.80 for AUC	The accuracy achieved needs improvement
[24]	Neelaveni and Devasana	Support vector machine (SVM) and decision tree (DT)	SVM achieved an accuracy of 85%.	The accuracy achieved needs improvement
[25]	Leong and Abdullah	Deep neural network (DNN), random forest (RF), gradient boosting machines (GBM), support vector machine (SVM), and logistic regression (LR)	RF achieved an accuracy, sensitivity, specificity, and AUC of 94.39%, 88.24%, 100.00%, and 94.44%, respectively	The sensitivity is considered low.
[26]	Wang et al.	Convolutional neural network (CNN)	CNN achieved an accuracy, sensitivity, and specificity of 97.65%, 97.96%, and 97.35%, respectively	The model is built using 8-layers which increases the required computational cost
[27]	Liu et al.	Linear SVC, Logistic Regression CV, decision tree (DT), bagging, and multiple layer perceptron (MLP)	Logistic Regression CV achieved precision, recall, F1 score, and accuracy of 87.5%, 91.3%, 89.4%, and 86.1%, respectively	The accuracy needs improvement
[28]	Almubark et al.	Random forest (RF), gradient boosting (GB), support vector machine (SVM), and adaptive boosting (AdaBoost)	SVM achieved an accuracy, specificity, and sensitivity of 91.08%, 94%, and 85.71%, respectively	The sensitivity is considered low
[29]	Revathi et al.	Support vector machine (SVM), random forest (RF), and multinomial logistic regression (LR)	SVM, RF, and multinomial LR achieved accuracy rates of 86%, 71%, and 89%, respectively	The accuracy is considered low
[30]	Goenka and Tiwari	3D- convolutional neural network (CNN)	The model achieved a testing accuracy of 100% with a loss of 12.74%, validation accuracy of 98.08% with a loss of 14.59%, training accuracy of 100% with a loss of 9.5%, and precision, recall, and F1 score of 100%	The 3D convolution layer increases the required computational cost

**Table 2:** Features description.

Feature	Description
Sex	Male or female
Age	Age in years
Temperature	The body temperature in degrees Celsius (C)
White blood cells (WBC)	The WBC count in a body
Red blood cells (RBC)	The RBC count in a body
Pulse ox	The measurement of oxygen in the blood (oxygen saturation)
Platelet	The platelet count in a body
MPV	The measurement of platelet size
RDW	The measurement of red cells size variance
MCH	The hemoglobin average in a single RBC
MCHC	The average quantity of hemoglobin in a single RBC per unit volume, considering the cell volume
MCV	The average size of RBCs
Hematocrit	The ratio of the volume of RBCs in the overall amount of blood
Hemoglobin	The measurement of hemoglobin level in the blood
Pulse	The number of heart beats per minute is also called the heart rate
Respiratory rate	The measurement of breathing rate per minute
BP-systolic	The highest blood pressure during ventricular contraction
BP-diastolic	The lowest pressure that is measured immediately before the subsequent contraction

**Table 3:** The statistical analysis of numerical attributes.

Features	Mean	STD	Min	25%	50%	75%	Max	Missing values
Age	55.79	20.58	11.00	37.00	59.00	74.00	92.00	0.00
Pulse	80.35	12.45	49.00	72.00	78.50	88.75	117.00	18.00
BP - systolic	121.15	18.66	51.00	110.00	120.00	135.00	172.00	19.00
Temperature	36.70	0.33	35.40	36.50	36.70	36.80	38.30	19.00
Respiratory rate	20.15	2.68	0.00	20.00	20.00	20.00	35.00	19.00
BP-diastolic	72.90	11.24	27.00	66.00	73.00	79.00	110.00	19.00
WBC	6.92	2.81	0.70	4.90	6.60	8.10	16.10	37.00
RBC	4.39	0.75	2.20	4.13	4.46	4.84	5.87	37.00
Hemoglobin	12.23	2.18	5.00	11.45	12.50	13.70	17.40	37.00
Hematocrit	36.83	6.49	14.20	34.00	37.60	40.55	50.80	37.00
MCV	84.26	7.70	59.10	80.05	85.00	89.50	99.70	37.00
MCH	27.89	2.81	17.50	26.55	28.10	30.00	33.80	37.00
MCHC	33.17	1.27	28.60	32.50	33.40	34.00	35.40	37.00
RDW	14.91	2.33	11.70	13.50	14.30	15.55	24.00	37.00
MPV	8.80	1.13	6.00	8.00	8.80	9.40	12.90	38.00
Platelet	238.65	82.46	5.00	186.25	243.00	289.75	517.00	38.00
Pulse ox	98.24	3.78	65.00	98.00	99.00	100.00	100.00	40.00

**Table 4:** The optimal hyperparameters of each classifier with the original and oversampled data.

Classifier	Hyperparameter	Without oversampling	With oversampling
SVM	Cost	5	4
	Gamma	1	1
	Kernel	Linear	RBF

K-NN	N_neighbors	5	5
	Metric	Minkowski	Minkowski

Adaboost	N_estimators	100	300
	Learning rate	0.1	0.1

XGBoost	N_estimators	100	500
	Booster	Gbtree	Gbtree
	Learning_rate	0.1	0.1

**Table 5:** Classifiers accuracy, precision, and recall using the optimal hyperparameters.

Classifier	Dataset	Accuracy (%)	Precision (%)	Recall (%)
SVM	Original	92.21	92.44	92.14
	Oversampled	93.33	93.32	95.56

k-NN	Original	87.63	86.54	84.29
	Oversampled	88.24	85.43	94.44

AdaBoost	Original	90.92	90.95	89.05
	Oversampled	91.16	92.32	92.22

XGBoost	Original	91.63	92.07	90.95
	Oversampled	91.60	91.42	93.33

**Table 6:** The best feature subset obtained for each classifier.

Classifier	Number of features	Features selected	Accuracy (%)
SVM	13	{Sex, age, pulse, respiratory rate, BP–diastolic, white blood cells, red blood cells, hemoglobin, hematocrit, MCV, MCH, RDW, MPV}	95.56
K-NN	6	{Sex, age, respiratory rate, hematocrit, MCH, RDW}	95.52
Adaboost	10	{Sex, age, BP–systolic, temperature, BP–diastolic, hematocrit, MCH, RDW, platelet, pulse ox}	95.00
XGBoost	6	{Sex, age, respiratory rate, white blood cells, MCV, MCHC}	94.38

**Table 7:** The performance of the final selected models.

Classifier	Accuracy (%)	Precision (%)	Recall (%)
SVM	95.56	94.70	97.78
k-NN	95.53	95.81	96.67
AdaBoost	95.00	96.00	94.44
XGBoost	94.38	94.18	95.56

## Data Availability

Alzheimer's disease clinical data used to support the findings of this study are available from the corresponding author upon request.
